# Multi-target mode of action of a Clerodane-type diterpenoid from *Polyalthia longifolia* targeting African trypanosomes

**DOI:** 10.1038/s41598-018-22908-3

**Published:** 2018-03-15

**Authors:** Godwin U. Ebiloma, Evangelos Katsoulis, John O. Igoli, Alexander I. Gray, Harry P. De Koning

**Affiliations:** 1 Institute of Infection, Immunity and Inflammation, College of Medical, Veterinary and Life Sciences, University of Glasgow, Glasgow, UK; 2 Department of Biochemistry, Faculty of Natural Sciences, Kogi State University, Anyigba, Nigeria; 3 Strathclyde Institute of Pharmacy and Biomedical Sciences, University of Strathclyde, Glasgow, UK; 4 Department of Chemistry, College of Science, University of Agriculture, Makurdi, Nigeria

## Abstract

Natural products have made remarkable contributions to drug discovery and therapy. In this work we exploited various biochemical approaches to investigate the mode of action of 16-α-hydroxy-cleroda-3,13 (14)-Z-dien-15,16-olide (HDK-20), which we recently isolated from *Polyalthia longifolia*, on *Trypanosoma brucei* bloodstream trypomastigotes. HDK20 at concentrations ≥ EC_50_ (0.4 μg/ml) was trypanocidal, with its effect irreversible after only a brief exposure time (<1 h). Fluorescence microscopic assessment of DNA configuration revealed severe cell cycle defects after 8 h of incubation with the compound, the equivalent of a single generation time. This was accompanied by DNA fragmentation as shown by Terminal deoxynucleotidyl transferase dUTP Nick-End Labelling (TUNEL) assays. HDK-20 also induced a fast and profound depolarisation of the parasites’ mitochondrial membrane potential and depleted intracellular ATP levels of *T*. *brucei*. Overall, HDK20 showed a multi-target mechanism of action, which provides a biochemical explanation for the promising anti-trypanosomatid activity in our previous report.

## Introduction

African trypanosomiasis is a disease caused by infection of humans and animals with various species or subspecies of trypanosomes, usually through the bite of infected tsetse flies, although some have adapted to other means of transmission and are no longer bound geographically to the tsetse habitat^1^. Unfortunately, most of the current drugs are ineffective due to drug resistance and efforts towards new drug development are inadequate^1,2^. Large numbers of synthetic chemo-types have been produced and tested on protozoan pathogens, yet have mostly failed to deliver new treatments for parasitic diseases. A particular problem remains with drugs against parasites causing the ‘most neglected tropical diseases’. Thus, most of the current drugs are old, and parasitic strains have become resistant to these drugs^3–5^. Unfortunately, the development of new and efficient anti-protozoan drugs has not received much attention or funding from the pharmaceutical industry for the reason that most of these diseases occur in resource-poor communities where the people suffering from these diseases cannot afford to pay the high price of the drugs, but rely heavily on subsidized or donated drugs^6^. Consequently, investing in drug development against especially tropical parasitic diseases is economically unattractive. The search for anti-parasitic drugs from natural sources, especially from secondary metabolites derived from plants, is a good alternative to synthetic drugs, considering that medicinal plants have been used for centuries for treating infectious diseases^7–9^.

**Figure 1:**
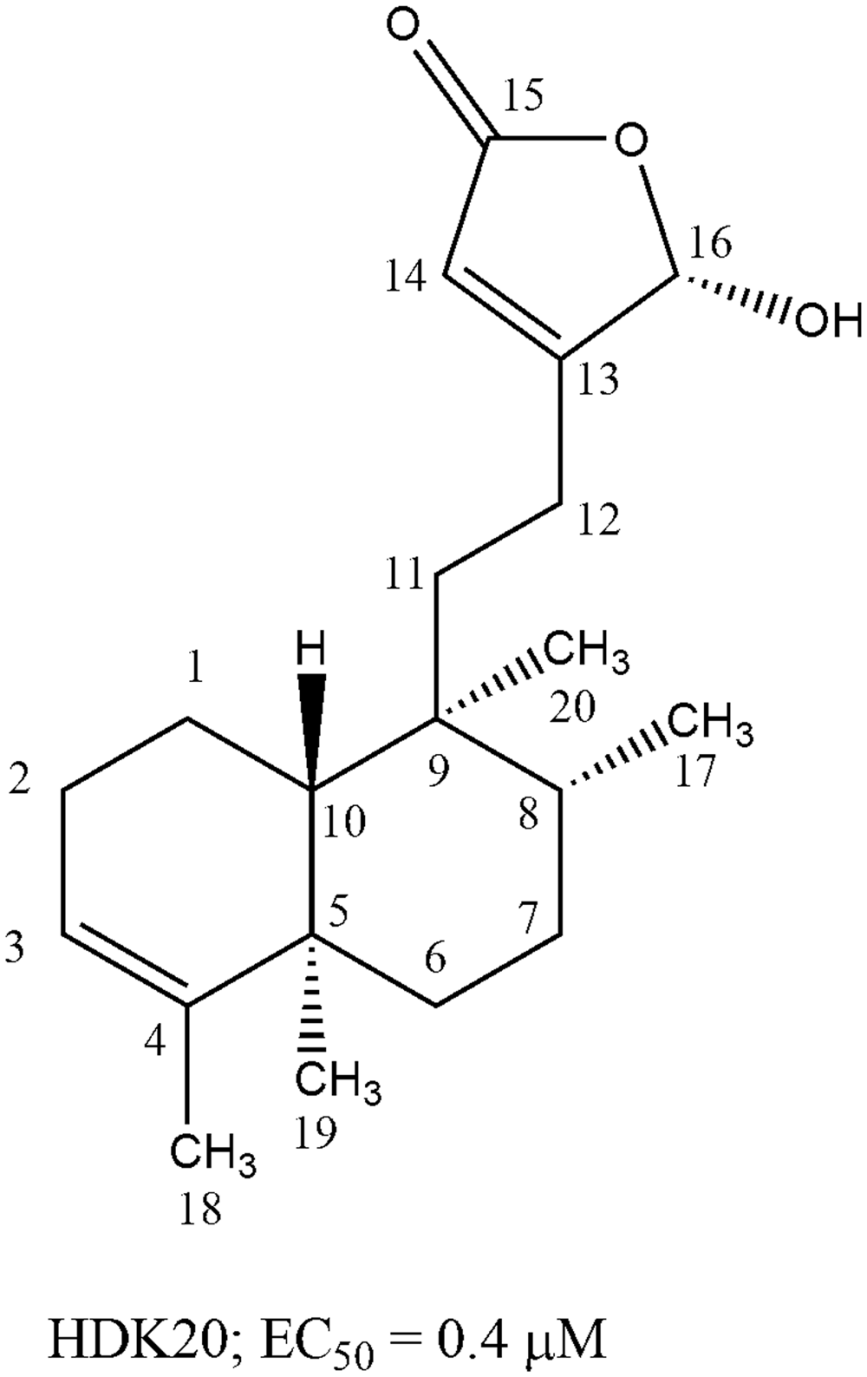
Structure of 16α-hydroxycleroda-3,13 (14)-Z-dien-15,16-olide, HDK20, showing *in vitro* activity against trypomastigotes of *T*. *brucei* as previously reported^12^.

Natural products continue to play a significant role in the development of new chemotherapies. For instance, in the years 1981 to 2006, 1,184 new drugs were developed and registered for use, out of which 28% were of natural origin. Another 24% of all the new drugs registered within this period had functional groups with pharmacological activity (pharmacophores) derived from natural products^10^. Although there have been many promising developments to identify leads against tropical protozoan parasites using various plant extracts, the translation of these results into actual drug candidates has not occurred. In most cases the active principles of these plant extracts remain unknown due to a lack of research facilities and funding. Moreover, the specific mechanism of action of these reported bioactive extracts or bioactive principles is rarely, if ever, determined (e.g., triptolide; curcumin)^11^. However, a comprehensive understanding of the interaction of a drug lead with its molecular target is especially helpful for the process of drug development, for the reason that it allows the use of medicinal chemistry approaches for optimization, and in some cases a more suitable clinical trial design. This is crucial for the development of new and efficient chemotherapy that will replace the current inefficient drugs. Here we report on a detailed investigation of the mode of action of a clerodane (16-α-hyroxy-cleroda-3,13 (14)-Z-dien-15,16-olide, HDK-20; Fig. 1) previously isolated from *Polyalthia longifolia*^12^ on *Trypanosoma brucei* trypomastigotes.

## Results

### Effect of different concentrations of HDK20 on the growth of bloodstream form *T*. *b*. *brucei* following long and limited exposures

**Figure 2:**
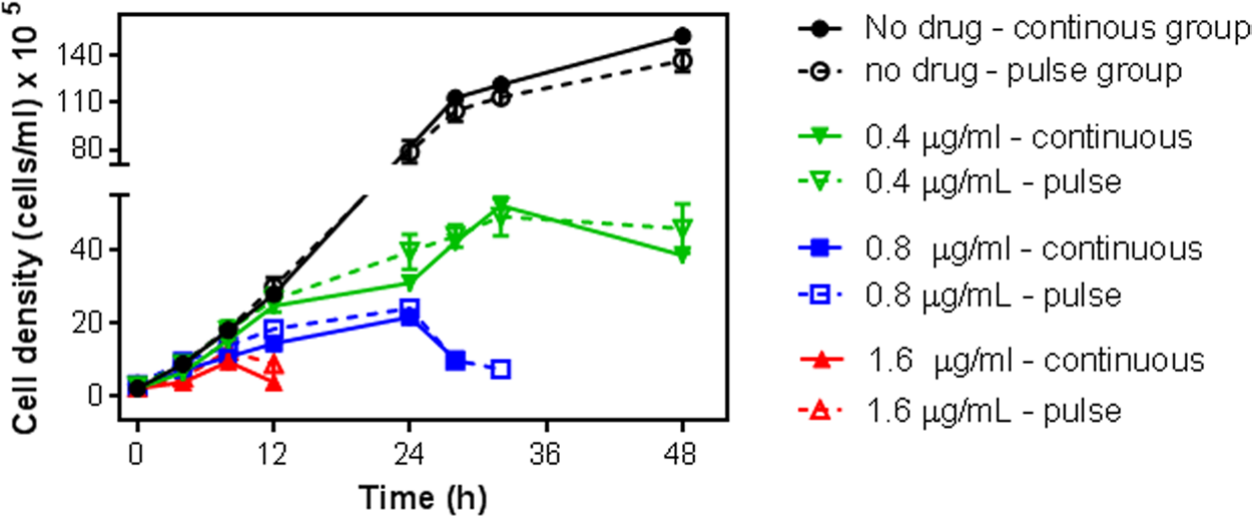
Growth curve of *T*. *brucei* grown in the continuous presence of HDK20 at indicated concentrations, or untreated (no drug) (solid symbols and lines), or after exposure to the same concentrations for 1 h only (open symbols, dashed lines). Seeding density was 2 × 10^5^ cells/mL, incubations were at 37 °C and 5% CO_2_. Values obtained were plotted against time using GraphPad Prism 5 software. The experiment was conducted over 48 h; no live cells could be detected microscopically in cultures with 4 × EC_50_ at the 24 h time point, and at the 36 h point in cultures treated with 2 × EC_50_. The data are the average and SEM of three independent experiments, each performed in triplicate; where no error bars are shown, they fall within the symbol. There were no significant differences between continuous and pulse data points for any of the concentrations tested (P > 0.05; unpaired Student’s t-test).

The effect of HDK20 on trypanosome proliferation was studied after both continuous and a limited-duration exposure. The result shows that the growth pattern of cells with long time exposure were very similar to those with a short exposure to the compounds (Fig. 2). At a concentration of 0.4 µg/mL, corresponding to 1 × EC_50_, the effect was to slow growth, particularly after 12 h; after 24 h the growth rate had slowed to almost zero, but microscopical observation revealed little evidence of cell death as the cells were intact and normally motile; the cell density became significantly different from their untreated controls at 24 h and beyond (P < 0.01; unpaired t-test). At 2 × EC_50_, the trypanostatic effect occurred earlier (P < 0.05 at 8 and 12 h for continuous and pulsed exposure, respectively), and the cell population started dying after 24 h; no live cells were detected after 28 and 32 h in the continuous and 1-h pulse exposure groups, respectively. In the 4 × EC_50_ group (1.6 µg/mL), the cell population was sterilized after 12 h, regardless whether the cells were exposed for 1 h or continuously to the compound (significantly different from control at 4 h (P < 0.01) and 8 h (P < 0.05) for continuous and pulsed exposure, respectively). These results show unambiguously that the effects of HDK20 on trypanosomes are irreversible after a limited exposure time of 1 h.

### Monitoring the action of HDK20 on trypanosomes using a propidium iodide based assay

The effects of varying concentrations of the test compound on membrane permeabilization were monitored over a 6-hour period using the fluorescent probe propidium iodide. This compound is excluded from trypanosomes unless there is a loss in plasma membrane integrity, usually brought on by a drug treatment, which allows entry, upon which the probe binds nucleic acids and emits a strong fluorescence signal^13^. Digitonin, which rapidly permeabilizes the cells, was used a positive control and indicates the level of fluorescence where all cells have become permeable to propidium iodide (PI).

**Figure 3:**
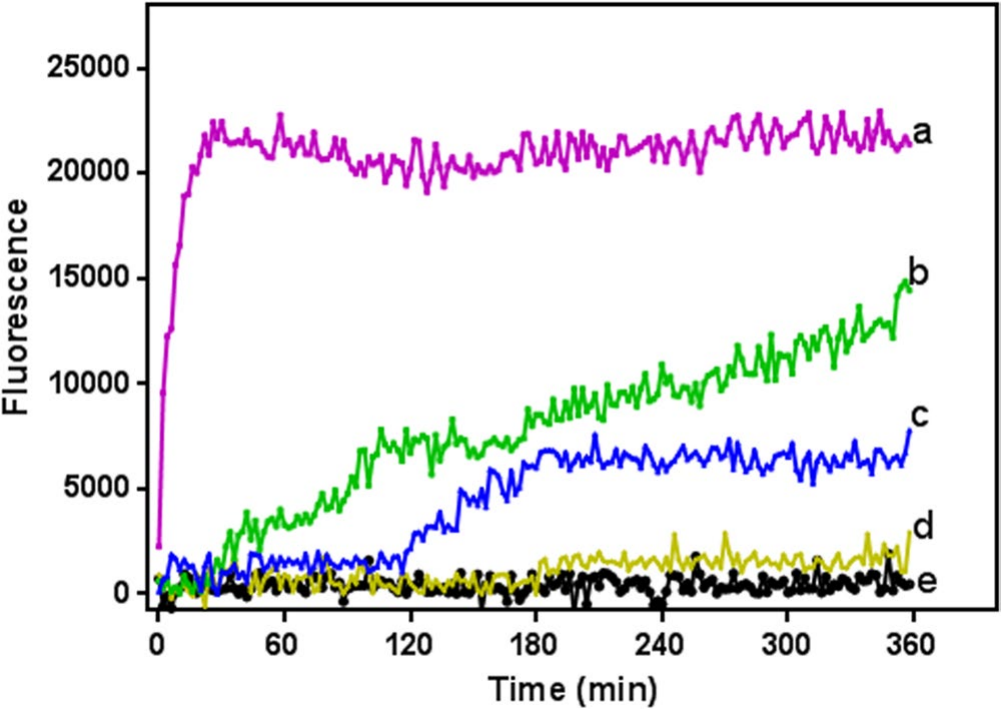
Results showing the speed of action of HDK20 on trypanosomes. Digitonin, a compound known to cause rapid cell permeabilization was used as positive control. Separate traces (background fluorescence were recorded in the absence of cells and these were subtracted from those presented herein. a = digitonin treated cells; b = 4 × EC_50_; c = 2 × EC_50_; d = cells + PI (untreated); e = media + PI (no cells).

Figure 3 shows that the compound led to a partial loss of plasma membrane integrity, which would start to affect cellular viability during the 6 hours of incubation, at concentrations of 2× and 4× their EC_50_ values. Trypanosomes treated with 0.8 μg/mL (2 × EC_50_) of HDK20 started becoming appreciably fluorescent after approximately 2–3 hours of incubation, but affecting only some 25% of cells during the 6-h period; however, when this concentration was doubled (1.6 µg/mL), the cells became fluorescent as early as 1–2 hours after the start of the incubation and the signal continued to increase to >50% by 6 h, indicating a steady increase in the number of cells with plasma membranes that had become permeable to PI. None of the treated groups reached the maximum fluorescence within the duration of the experiment as indicated by the digitonin-treated control cells. All this was consistent with the growth curves depicted in Fig. 2. Yet, the growth curve showed clear effects on population growth rates, with trypanostatic effects at 1–2 × EC_50_ that are not easily explained in terms of the disintegration of the plasma membrane, and we conclude that the cell membrane permeabilization visualized in Fig. 3 as PI fluorescence is the *result* of cell death, rather than that being the *cause* of cell death.

### HDK20 causes cell cycle arrest in trypanosomes

Some compounds purified from natural sources elicit their activity by inhibiting progression of the cell cycle, for instance by binding to a protein required for cell division, or by intercalating with DNA. Thus, an understanding of cell cycle events may help in deciphering the mode of action for HDK20, especially given the effects on *in vitro* growth at 1–2 × EC_50_, and we assessed the effect of low, non-lethal concentrations of this test compound on cell cycle progression in trypanosomes.

**Figure 4:**
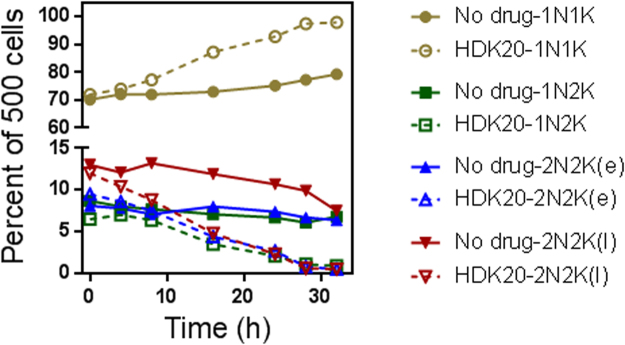
Cell cycle determination in *T*. *b*. s427 WT using DAPI. Fixed cells prepared on slides were viewed under a fluorescence microscope after up to 32 h of incubation with 0.2 µg/mL HDK20 (open symbols, dashed lines); the untreated control culture (no drug; closed symbols, solid lines) was grown in parallel. Values were expressed as percentage of 500 counted cells. N = nucleus; K = kinetoplast; e = early stage; L = late stage with initiated cytokinesis. Sample micrographs of each cell division stage are provided in Supplementary Figure S1.

Examination of DNA configuration by fluorescence microscopy with the DNA stain DAPI revealed progressive cell cycle defects from 8 hours of incubation with just 0.2 µg/mL HDK20 (0.5 × EC_50_). Under these conditions, kinetoplast division could not be completed or perhaps not initiated, leading to a dramatic increase in the percentage of cells in G1/S phase, with one nucleus (1 N) and one kinetoplast (1 K), and a concomitant decrease in cells in all stages of the cell division process (Fig. 4), leading to the nearly complete disappearance of dividing cells from the treated populations, while the proportions of 1N1K and 2N2K cells remained constant in the non-treated population grown in parallel. This result showed that the (initiation of) DNA synthesis, particularly of kinetoplast DNA (kDNA), might be a target for the trypanocidal activity of the test compound. The apparent G1 or S-phase cell cycle arrests induced by the continuous exposure to low concentrations of the compound for up to 30 h are consistent with the growth inhibition depicted in Fig. 2.

### Microscopical investigation of cellular morphology of *T*. *brucei* treated with HDK20

Fluorescence microscopy after DAPI staining showed that 70% of the cell population incubated with HDK-20 (0.4 µg/ml) contained one nucleus and one kinetoplast (1N1K) DNA after 8 hours of incubation (Supplementary Figure S1), consistent with the observation, made above, that HDK20 causes cell cycle arrest of *T*. *brucei* in G1 phase. Although the mitochondrion and kinetoplast of the cells remained apparently normal throughout the experiment, in some cells treated with HDK20 the nucleus appeared to have fragmented, with multiple nuclear fragments but only 1 kinetoplast (Supplementary Figure S1); this was not observed in the untreated control cells, throughout the period of the experiment.

### Assessment of DNA breaks using TUNEL assay

**Figure 5:**
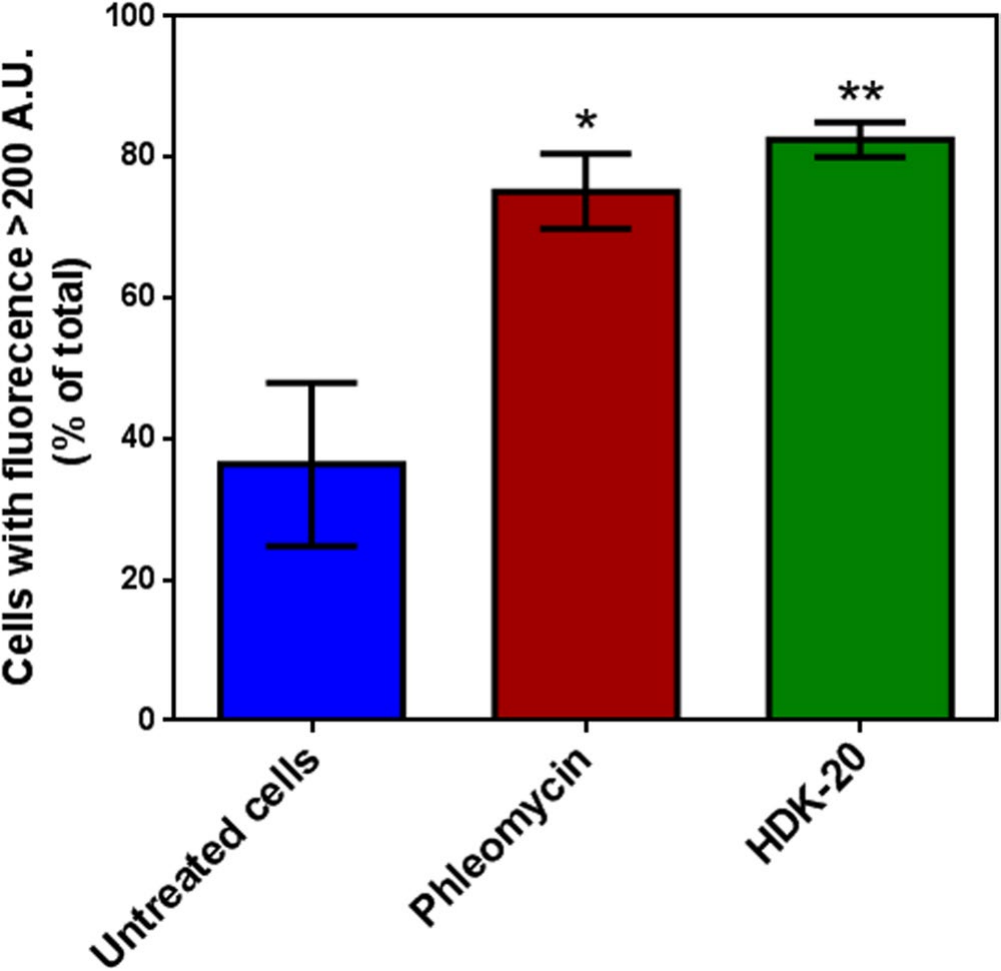
DNA fragmentation of treated and untreated *T*. *brucei s427* cells following 12 hours of incubation with the test compound (HDK20, 0.4 µg/mL) or without the test compound (untreated cells), and with phleomycin (2 μg/ml; positive control. This result is a summary of the histogram plots presented in Supplementary Figure S2. *p < 0.05; **p < 0.01 (n = 3). A.U., arbitrary units of fluorescence.

Cell cycle defects may lead to apoptosis or programmed cell death, and the latter is known to be linked with DNA fragmentation. Conversely, cell cycle arrest may be the result of DNA damage. Consequently, a TUNEL assay was used to study DNA fragmentation upon incubation with 0.4 µg/mL HDK20. Br-dU fluorescence was strongly increased in cells after 12 hours incubation with HDK20 (P < 0.01), slightly higher than the positive control, cells treated with the antibiotic phleomycin, which is known to cause double-strand breaks^14^ including in *T*. *brucei*^15,16^ (P < 0.05) (Fig. 5, Supplementary Figure S2).

### Effects of HDK-20 on mitochondrial membrane potential of *T*. *b*. *brucei* treated cells

HDK20 affected the cell cycle progression of *T*. *brucei* cells in this study, leading to an apparent accumulation of cells in the G_1_ phase of the cell cycle and the accumulation of 1N1K cells. A direct causal effect has been previously reported between the mitochondria membrane potential and cell cycle progression in *T*. *brucei*^17^. Consequently, we examined whether this compound affects the mitochondrial function in bloodstream trypanosomes and whether this occurs before or after the cell cycle defects observed earlier.

**Figure 6:**
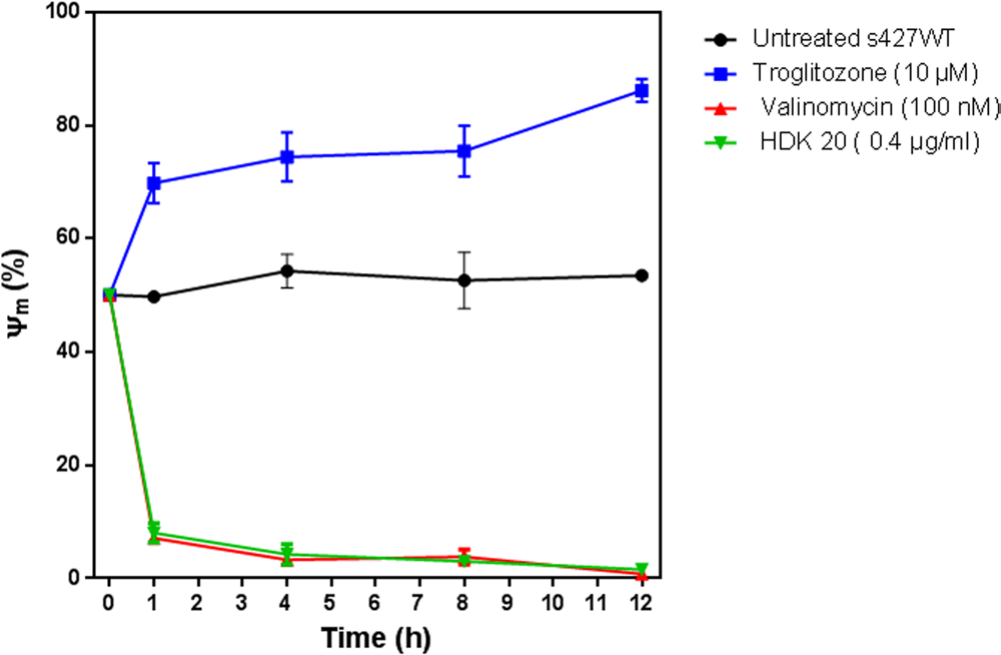
Mitochondrial membrane potential (ΔΨm) of treated and untreated *T*. *b*. *brucei* bloodstream forms. ΔΨm was determined with a flow cytometer using *T*. *b*. *brucei* (bloodstream-form) incubated with 25 nM tetra-methylrhodamine (TMRE), and expressed as the percentage of cells exhibiting >200 A.U. Troglitazone (10 µM) was used as mitochondrial membrane hyperpolarization control, while valinomycin (100 nM) was used as mitochondrial membrane depolarization control. A shift to a higher fluorescence indicates hyperpolarisation (increased ΔΨm); while a shift to a lower fluorescence is indicative of a decreased ΔΨm (depolarization) (See Figure S3).

Figure 6 presents a summary of the mitochondrial potential (ΔΨm) changes during 12 h of incubation with or without the test compound and read at 0, 1, 4, 8 and 12 h. As expected, there was no change in the ΔΨm for untreated cells for the duration of the experiment (12 h), whereas valinomycin induced a very rapid and strong depolarisation of the ΔΨm after just 1 h of incubation, and troglitazone caused a clear hyperpolarisation of the mitochondrial membrane. HDK-20, even at a relatively low concentration (0.4 µg/mL) induced a rapid and profound depolarisation of the parasites’ mitochondrial membrane potential at the first time point (1 h of incubation) and the trend continued until a near complete depolarization was achieved after 12 hours. The flow cytometry histograms are provided as Supplementary Figure S3. This result indicates that HDK20 induced a rapid depolarisation of mitochondrial membrane potential as an early effect on the parasites.

### Effects of HDK20 on ATP levels in *T*. *b*. *brucei*

The ΔΨm experiment revealed that HDK20 caused depolarisation of the mitochondrial membrane of *T*. *brucei*, which in bloodstream forms is maintained by the oligomycin-sensitive F_o_F_1_ ATP synthase^18^. One possibility is that a reduction in cellular ATP levels leads to the reduced mitochondrial membrane potential by interfering with ATP production (or the mitochondrial ADP/ATP exchanger), thereby denying the F_1_F_o_-ATPase the ATP it needs to pump H^+^ across the inner mitochondrial membrane. In that scenario, one would expect to see a dramatic reduction in cellular ATP levels preceding the rapid decline in ΔΨm. In order to assess this possibility, we tested the effect of HDK20 on ATP production, at the same concentration and over the same period as assessed on ΔΨm.

**Figure 7:**
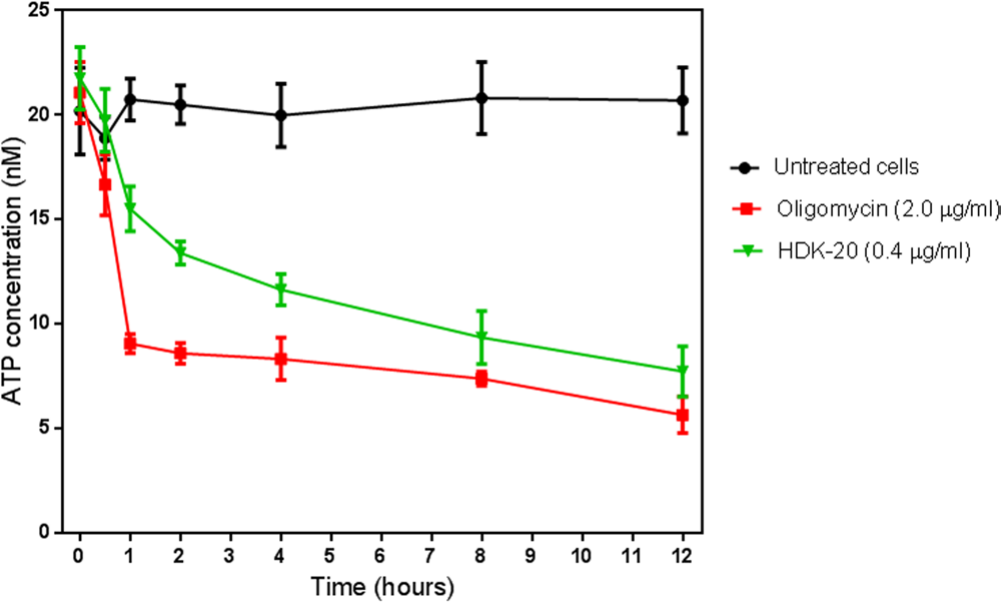
ATP concentrations in 1 × 10^7^ treated and untreated *T*. *brucei* cells. Incubation was for a period of 12 hours, with samples taken at each indicated time point. The results shown here are the averages and SEM of four fully independent experiments.

Intracellular ATP levels of *T*. *brucei* treated with HDK20 were found to be severely depleted, but only after several hours of incubation (Fig. 7). Although the intracellular ATP level in the cells treated with HDK20 trended down after just one hour of incubation, this only became statistically significant after 2 hours of incubation with the test compound. Oligomycin was used as a positive control as it specifically inhibits the *T*. *brucei* F_1_F_o-_ATPase, which in bloodstream forms pumps protons out across the inner mitochondrial membrane^18^; oligomycin is known to reduce ATP levels in bloodstream trypanosomes by 50%^17,19^, and if HDK20 likewise acts directly on this ATPase it should mirror the actions of this inhibitor. The fact that oligomycin induced a much more rapid depletion of ATP, compared to that exhibited by HDK20 (at a concentration that rapidly depolarised the mitochondrial membrane (Fig. 6)), seems to indicate that this test compound does not, in fact, directly inhibit the F_1_F_o_-ATPase. Moreover, the gradual decline in ATP content clearly shows that HDK20 first depolarises the inner mitochondrial membrane, which subsequently impacts on ATP production.

## Discussion

We have previously reported the promising activity of HDK20 against *T*. *brucei*^12^ and here report an investigation of the cellular and biochemical effects this natural compound, isolated from the Nigerian medicinal plant *Polyalthia longifolia*^12^, exerts on the parasites. We observed that the compound rapidly (within 1 h) and irreversibly induced cell cycle arrest in G1 phase, followed by cell death at concentrations below 1 µg/mL; this was accompanied by double strand DNA breaks. In that first hour of incubation, the compound caused a strong depolarization of the mitochondrial membrane, leading to a slower decline in ATP levels as mitochondrial function deteriorated.

The G1 phase cell cycle arrest, leading to 100% of the cell population with 1 nucleus and 1 kinetoplast after incubation with 0.2 µg/mL HDK20 (0.5 × EC_50_), was not simply the result of the reduction in ATP levels, which reached approximately 50% after 8 h, as the dividing cells appeared to complete their cell cycles: there was no accumulation of cells in any stage of the cell division process. Instead, there was an apparent failure to initiate a new cell division cycle, specifically the failure to replicate the kinetoplast, as the number of 1N2K cells rapidly declined upon treatment with HDK20 (Fig. 4). The conclusion that already initiated cell division was completed seems to explain the slight increase in cell density observed in cells incubated with concentrations as high as 0.8 µg/mL or 1.6 µg/mL HDK20 over the first few hours (Fig. 2).

In addition, HDK20 has previously been shown to be able to inhibit recombinant *Leishmania donovani* Topoisomerase I^20^, which could certainly contribute to the appearance of double strand breaks, if not to the very rapid effect on ΔΨm. The related compound clerocidin reportedly inhibits Topoisomerase II in cancer cells, leading to an accumulation of cells in G2/M phase and only 2% of cells in G1 phase after 17 h of incubation with the compound^21^ – in sharp contrast to the near-100% accumulation in G1 phase reported here after incubation of ≥24 h with HDK-20, which is not indicative of a principal action through topoisomerase inhibition. We thus propose that the effects on ΔΨm are directly responsible for the cell cycle effects, which starts with the replication of the flagellar basal body and the kinetoplast^22^, and that inhibition of topoisomerases^20,21^ may be responsible for the observed DNA fragmentation in the TUNEL assay and also plays a role in a multi-target activity against trypanosomes.

The kinetoplast of trypanosomatids is a structure of mitochondrial DNA that is highly organised, and is made up of tens of maxicircles and thousands of minicircles^23^. To ensure that *T*. *brucei* daughter cells receive identical kDNA networks, the process of replication is highly synchronized with replication of the nuclear materials, prior to cell division; thus, the mitochondrial S-phase for kDNA replication is initiated before the longer nuclear S-phase^22,24^. Indeed, the initiation of mitochondrial division appears to be an essential checkpoint that must precede nuclear division, leading necessarily to the creation of 1N2K cells before the onset of nuclear division and mitosis^22,25^. The here observed accumulation of 1N1K cells thus appears to indicate a failure to either initiate or complete the mitochondrial S-phase, i.e. kDNA replication. With the mitochondrial S-phase not being concluded, the nuclear S-phase would not commence, resulting in the observed cell cycle arrest at 1N1K.

Replication of the kDNA is dependent on intracellular ATP and hundreds of mitochondrial proteins^26^, which are imported into the mitochondria^27^ by a process that is dependent on both ATP and ΔΨm^28^. It is therefore reasonable to conjecture that the severe depletion of ATP and the decrease of the parasites’ mitochondrial membrane potential interfered with the successful initiation or conclusion of kDNA replication, resulting in cell cycle arrest due to the failure of cells to enter (or progress from) the kinetoplast G1 phase.

In conclusion, the mode of action of HDK20 against *T*. *brucei* appears to be multi-targeted, inflicting irreversible cell cycle arrest on trypanosomes. Additional mode of action studies, an investigation of the Structure Activity Relationship (SAR) and analysis of related compound libraries could further improve the efficacy of this compound. Importantly, HDK20 has been reported to be non-toxic and orally active, displaying promising activity in a hamster model of visceral leishmaniasis^20^.

## Materials and Methods

### Trypanosome strains and cultures

All experiments were performed with bloodstream trypomastigotes of *T*. *b*. *brucei* strain Lister 427, grown in culture exactly as described, in Hirumi’s Modified Iscove’s medium 9 (HMI-9; Life Technologies) supplemented with 10% heat-inactivated Fetal Calf Serum (FCS; Biosera), 14 μL/L β-mercaptoethanol (Sigma), and 3.0 g/L sodium hydrogen carbonate (Sigma) per litre of medium, pH 7.4 (complete HMI-9) at 37 °C in a 5% CO_2_ atmosphere^29^.

### Effect of different concentrations of HDK20 on the growth of bloodstream form *T*. *b*. *brucei* following long and time-limited exposures

Varying concentrations of the test compound were tested on trypanosomes for determination of *in vitro* cell growth using cell counts. Trypanosomes at their mid logarithmic phase of growth were taken from cultures and the cell density, determined with a haemocytometer, was adjusted to 2 × 10^5^ cells/mL with fresh complete HMI-9 medium. After predetermined periods of exposure to the test compound, cell counts were taken in triplicate, for each concentration of the compound, typically for up to 32 h. The experiment was repeated two more times and the counts of the three independent determinations were averaged and used for plotting the growth curve. For the time-limited exposure time experiments, the initial seeding density was 2 × 10^5^ cells/mL, the cells were treated with the test compound at varying concentrations except for the drug-free control. The cultures were first incubated at 37 °C and 5% CO_2_ for a brief period of 1 hour, after which the cells were washed twice with fresh media by centrifuging at 1200 RCF, re-seeded (2 × 10^5^ cells/mL) in fresh HMI-9 media, and samples were taken and counted at each time point. The counting was performed in triplicate and the average values obtained were plotted against time using Prism 5 software (GraphPad).

### Drug sensitivity assay using Propidium Iodide (PI)

Propidium iodide (Sigma-Aldrich) was used to monitor the effect of the test compound on trypanosome survival in real time^30,31^. Trypanosomes become fluorescent when the plasma membrane is breached and PI enters the cell and binds to nucleic acids^13^. For this assay, 100 μL of complete HMI-9 was added to 2 wells of a 96-well plate and a further 100 μL of complete HMI-9 but containing 3.2 µg/ml of HDK20, was added to the first well; 100 μL from this well (1.6 µg/ml HDK20) was then added to the second well to achieve a 0.8 µg/ml solution, after which 100 μL was removed from the second well and discarded. A third well received 100 µl of 20 µM digitonin in HMI-9, to serve as positive control for fluorescence when all cells rapidly lyse, and a fourth well contained HMI-9 medium only, to serve as negative control. To each well was added 100 µL of complete HMI-9 containing 2 × 10^6^ trypanosomes and 18 μM of PI. A final well containing the same final concentration of PI (9 µM) in complete HMI-9 but no cells served to record background fluorescence in the absence of cells, which was subtracted from the other traces. The plates were incubated in a FLUOstar OPTIMA fluorimeter (BMG Labtech) at 37 °C with 5% CO_2_ atmosphere, and the fluorescence was recorded at 544 nm excitation and 620 nm emission for 6 hours.

### Determination of mitochondrial membrane potential (ΔΨm) using flow cytometry

Changes in the mitochondrial membrane potential (ΔΨm) due to exposure of trypanosomes to the test compound were determined using flow cytometry after loading the cells with tetramethylrhodamine ethyl ester (TMRE; Sigma-Aldrich)^32,33^. The cell density was adjusted to 1 × 10^6^ cells/mL with and without test compound for the start of the experiment. 1 mL of sample was transferred at each time point into a microfuge tube and centrifuged at 1200 RCF for 10 min at 4 °C. The pellet was re-suspended in 1 mL PBS containing 200 nM of TMRE, followed by incubation at 37 °C for 30 min. The suspension was placed on ice for at least 30 min before analysis by a Becton Dickinson FACS Calibur using a FL2-heigth detector and CellQuest, and FlowJo software^30^. Valinomycin (100 nM; Sigma-aldrich) and troglitazone (10 μM; Biomol) were employed as negative (mitochondrial membrane depolarization) and positive (mitochondrial membrane hyperpolarization) controls respectively^30,32^. ΔΨm was determined at 0, 1, 4, 8 and 12 h.

### Assessment of mitochondrial integrity and DNA configuration (cell cycle) using fluorescence microscopy

Cells were incubated for various times in the presence and absence of HDK20, washed twice in filter-sterilized 1× PBS, spun at 2,600 rpm for 10 minutes and re-suspended in 1 mL of 1× PBS. 50 μl of the re-suspended cells were spread onto a glass microscope slide, and were left to air dry, the cells were then fixed in 1 mL of 70% methanol in PBS overnight at 4 °C. The mitochondrial integrity was examined by incubating 5 × 10^5^ cells/mL with 100 nM Mito-Tracker Orange CMTMRos fluorescent dye (ThermoFisher Scientific, UK)^34^. The configuration of nuclei and kinetoplasts of treated and untreated *T*. *brucei* was visualized using the fluorescent DNA dye 4,6-diamidino-2-phenylindole (DAPI) (Vector Laboratories, CA, USA), using a Zeiss Axioplan microscope as described^15^. A total of 500 cells per slide were counted for each sample, and scores for DNA configuration were given according to these groups: 1N1K, 1N2K, 2N2K (Early) and 2N2K (Late), where N is nuclear DNA; and K is kinetoplast DNA; Early and Late refer to the absence or presence, respectively, of an ingression furrow as indicator of cytokinesis in the cell division process of trypanosomes.

### Monitoring DNA fragmentation using TUNEL assay

Fragmentation of parasite DNA after exposure with the test compound was evaluated by TUNEL assay using the APO-BrdU TUNEL Assay Kit (Invitrogen) for the detection of DNA fragmentation caused by the effect of drug treatment, and utilizes 5-Bromo-2′-Deoxyuridine 5′-Triphosphate (BrdUTP) and Terminal deoxynucleotidyl transferase (TdT) enzyme. This was carried out following the manufacturer’s protocol, and exactly as previously described^16^. The treated samples were analysed by flow cytometry using both FL3- width and Anti-BrdU FITC detectors and cell Quest software. The data obtained was analysed with FlowJo 10 software.

### ATP level determination in BSF *T*. *brucei* using luciferase

Intracellular ATP was measured using an ATP Determination Kit (Invitrogen), which contains recombinant firefly Luciferase and Luciferin, following the manufacturer’s protocol. Briefly, cultures containing 1 × 10^7^ cells of both treated and untreated parasites were taken and centrifuged for 10 min at 1200 × *g* (4 °C). The resulting pellets were washed twice in 200 μL of 50 mM Tris-HCl, pH 7.4, containing 0.1 mM Dithiothreitol (DTT). The cells were then lysed by sonication on ice (10 seconds twice, separated by 30 s) using a Soniprep 150 with amplitude set at 8 μm. After this, it was centrifuged at 17,000 × *g* for 10 min at 4 °C. The resultant supernatant was frozen in liquid nitrogen and stored at −80 °C. Cellular ATP levels were measured and quantified using a serially diluted standard (0.5 μM–500 pM) following the manufacturer’s protocol, measuring luminescence with a FLUOstar OPTIMA plate reader.

### Data availability

All data generated or analysed during this study are included in this published article (and its Supplementary Information files).

## Electronic supplementary material

Supplementary information
