# Supplementary material for: Multi-target mode of action of a Clerodane-type diterpenoid from Polyalthia longifolia targeting African trypanosomes
Source: Sci Rep. 2018 Mar 15;8:4613. doi: 10.1038/s41598-018-22908-3 (PMC5854603; doi:10.1038/s41598-018-22908-3)
Supplement: Supplementary file 1 — Supplementary information [file 41598_2018_22908_MOESM1_ESM.doc]

**Supplementary file.**

**Multi-target mode-of-action of a Clerodane-type diterpenoid from *Polyalthia longifolia* targeting African trypanosomes.**

Godwin Unekwuojo Ebiloma^1,2^, Evangelos Katsoulis^1^, John Ogbaji Igoli^3,4^, Alexander Ian Gray^4^ and ^*^Harry P. De Koning.^1^

1 Institute of Infection, Immunity and Inflammation, University of Glasgow.

2 Department of Biochemistry, Faculty of Natural Sciences, Kogi State University, Nigeria.

3 Department of Chemistry, College of Science, University of Agriculture, Makurdi, Nigeria.

4 Strathclyde Institute of Pharmacy and Biomedical Sciences, University of Strathclyde.

* Author for correspondence:

Harry P. de Koning

Institute of Infection, Immunity and Inflammation
College of Medical, Veterinary and Life Sciences
University of Glasgow.
Sir Graeme Davies Building 
120 University Place
G12 8TA
Glasgow, Scotland
United Kingdom
Tel (+44) (0)141-330 3753
Email: Harry.de-Koning@glasgow.ac.uk

|  | **bright field** | **DAPI** | **Mitotracker** | **Merge** |
| --- | --- | --- | --- | --- |
| 1N1K  (untreated) | **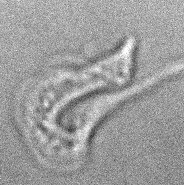** | **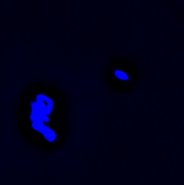** | **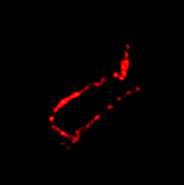** | **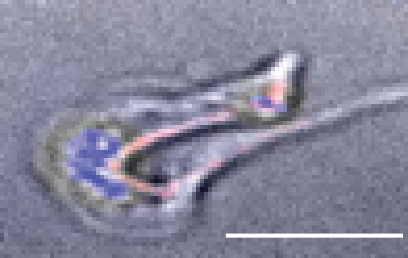** |
| 1N2K  (untreated) | **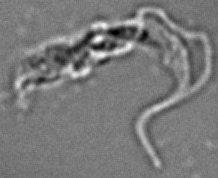** | **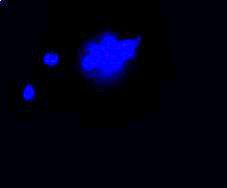** | **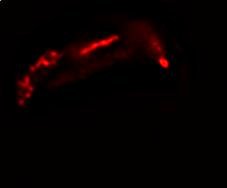** | **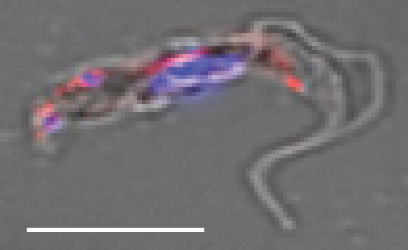** |
| 2N2K-E  (untreated) | **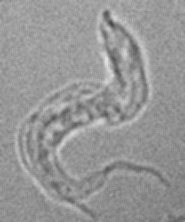** | **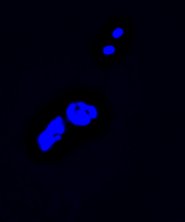** | **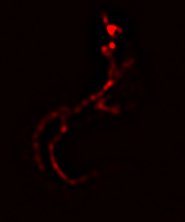** | **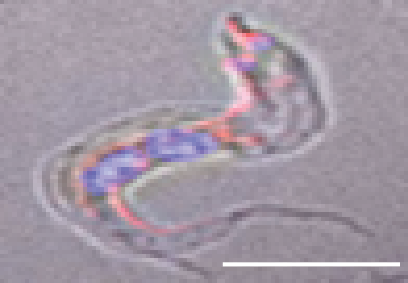** |
| 2N2K-L  (untreated) | **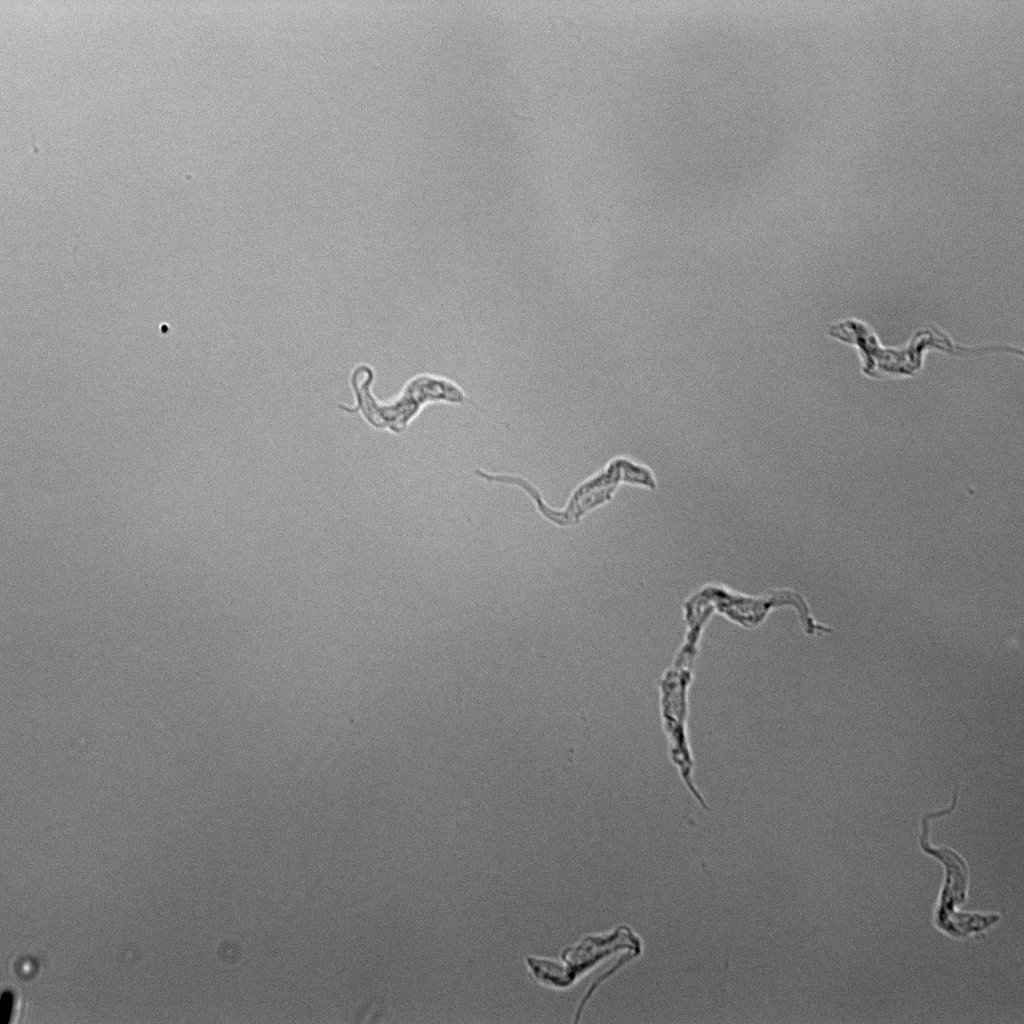** | **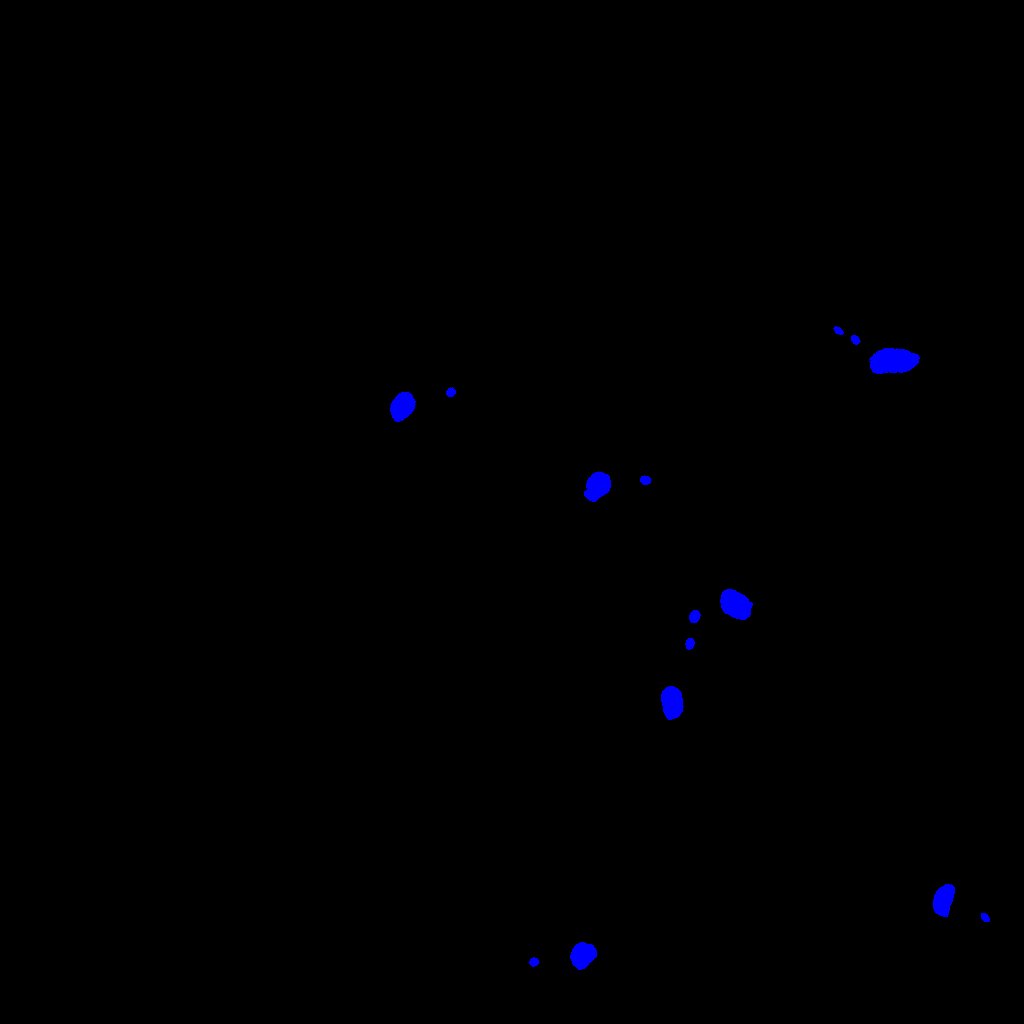** | **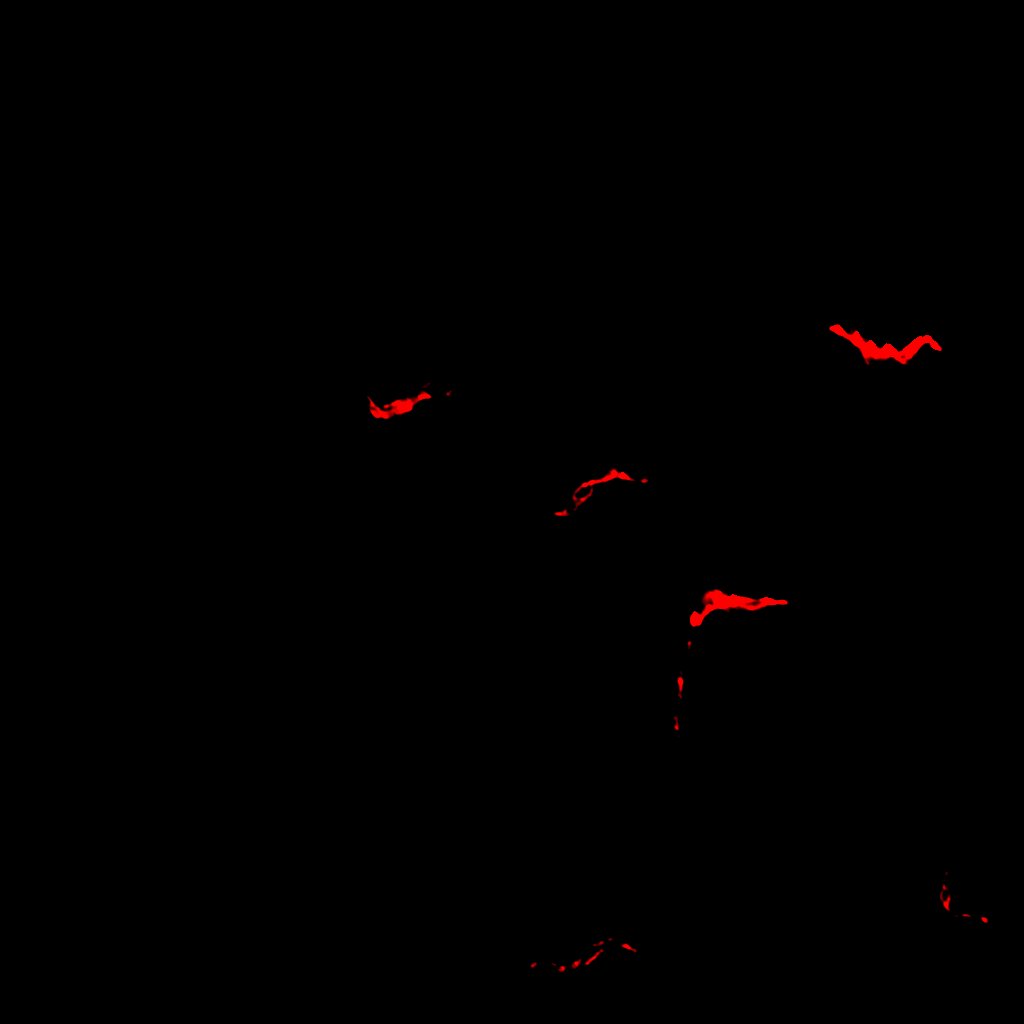** | **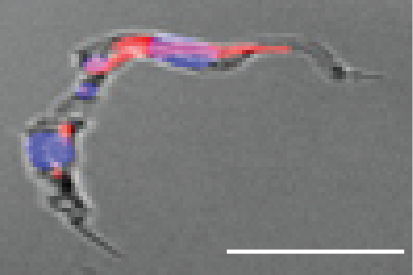** |
| HDK20-treated cells (**8 h**). All  1N1K. | 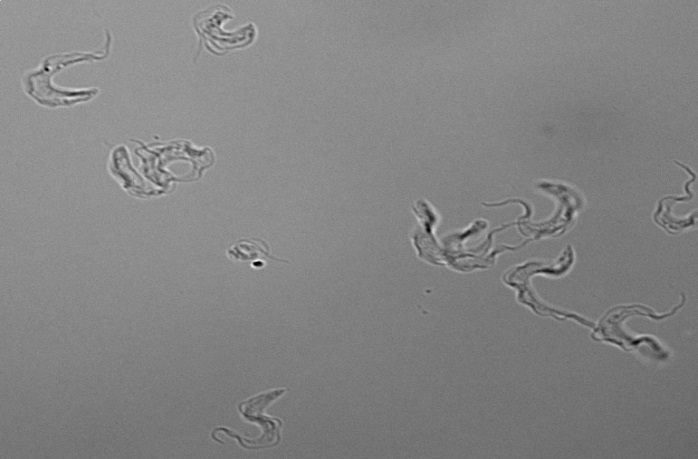 | 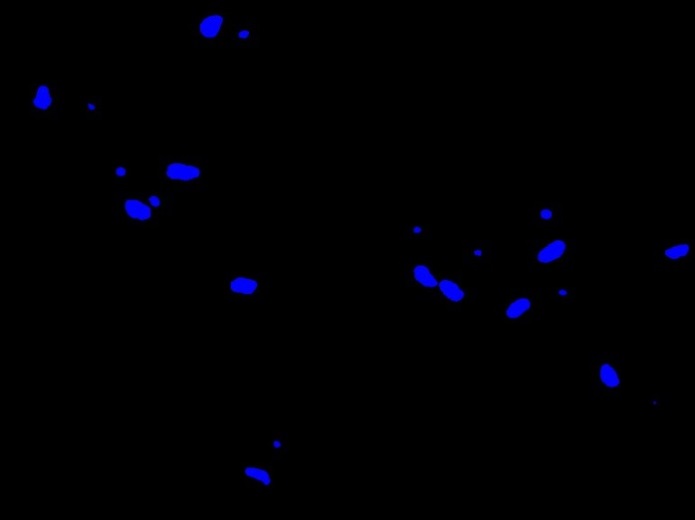 | 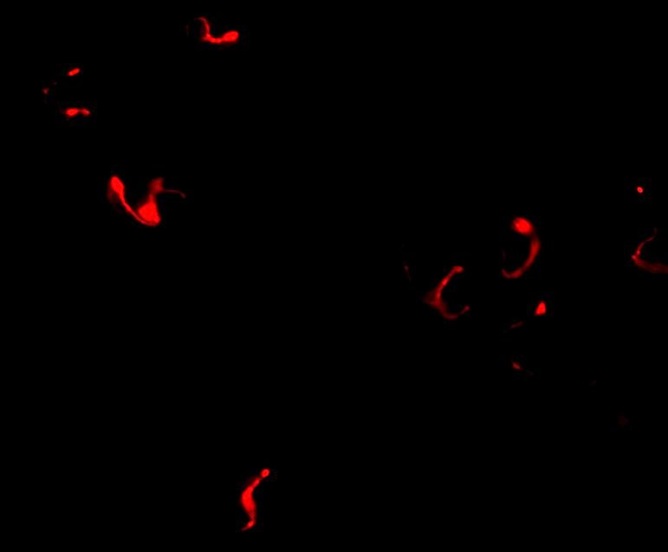 | 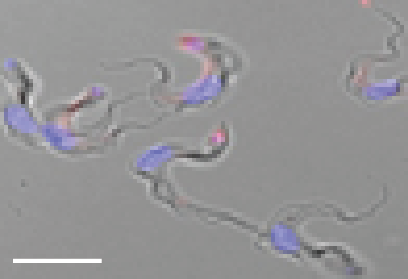 |
| HDK20-treated cells (**10 h**)  DNA breaks? | 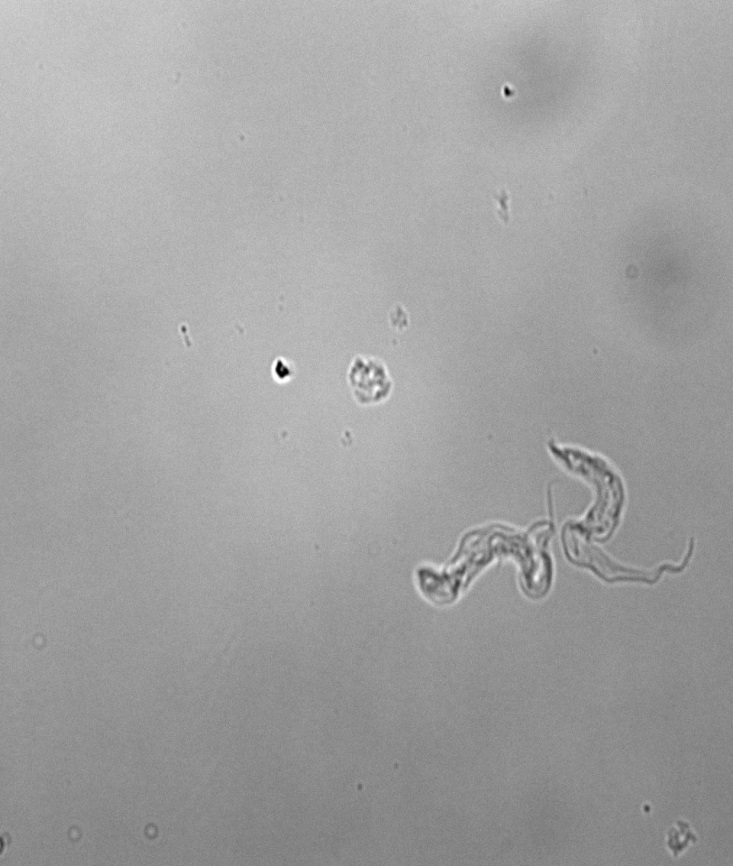 | 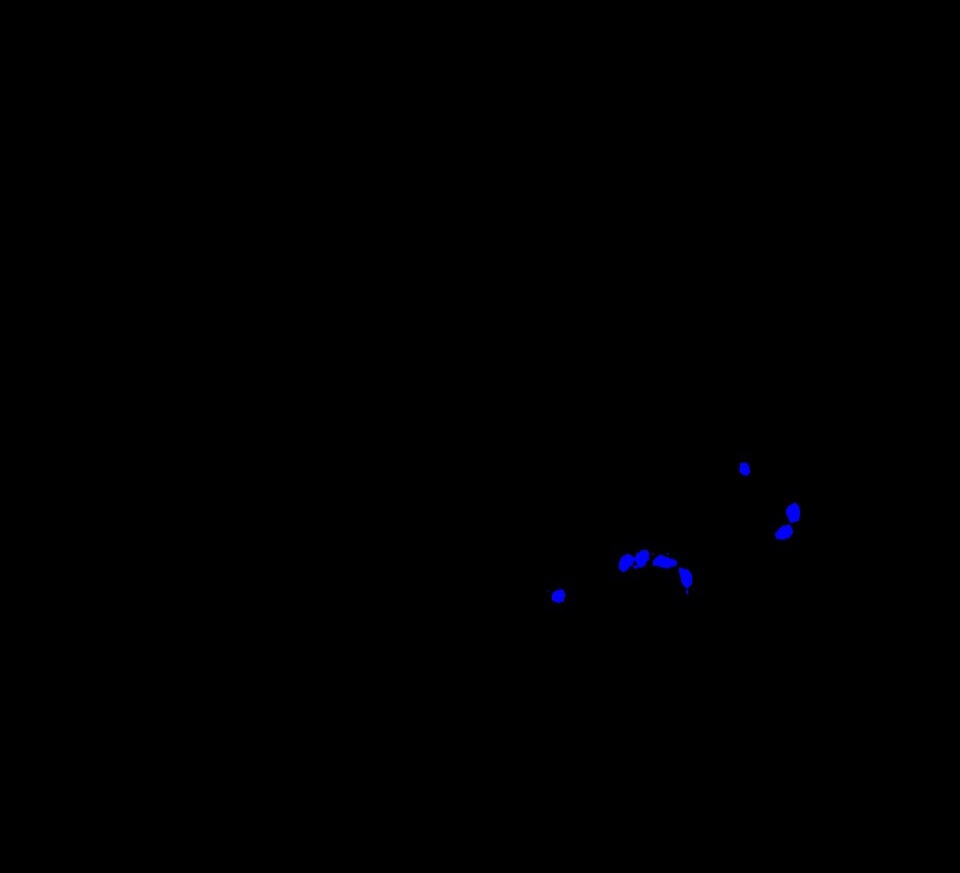 | 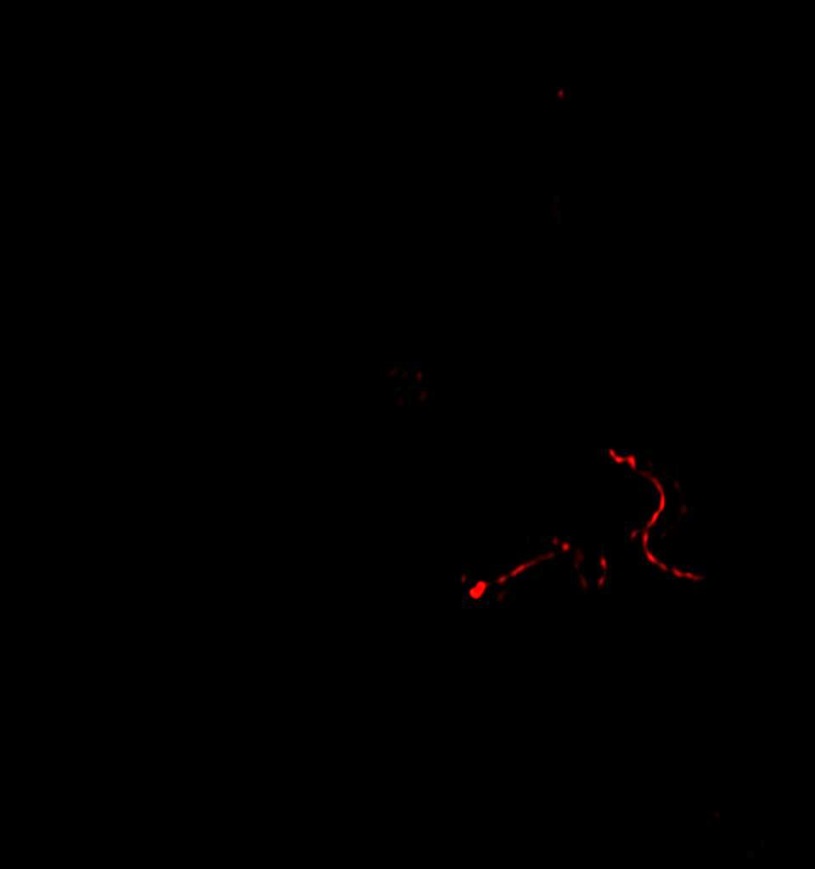 | 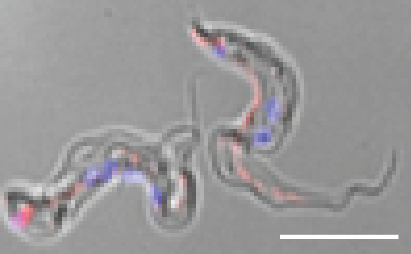 |

**Figure S1.** DNA contents and mitochondrial examination of treated and untreated (ND) cells. Bars: 5 µm.

Row 1-4: examples of untreated cells in various stages of cell division as indicated. Frames show single cells to allow optimal visibility of the salient features of these cell division stages.

Row 5. Treated cell population had 1N1K after 8 hours of incubation. Frame shows multiple cells (6) to indicate that the population rather than a single cell is at 1N1K stage.

Row 6. Images of *T. brucei* s427 wild type showing apparent nuclear fragmentation following 10 hours of incubation with the test compound (HDK20, 0.4 µg/ml). Again, more than one cell is shown, in order to indicate that this is not an isolated occurrence. The distribution of nuclear DNA is not compatible with normal cell division, as in those cases the maximum number of nuclei is 2 and at most equals the number of kinetoplasts, as illustrated in rows 1-4; moreover, the cells shown do not appear to undergo cytokinesis.

N = nuclear DNA; K = kinetoplast DNA; e = early stage; L = late stage; DAPI = 4',6-diamidino-2-phenylindole.

| **Samples** | **Experiment 1** | **Experiment 2** | **Experiment 3** |
| --- | --- | --- | --- |
| **Drug free** |  |  |  |
| Phleomycin  (2 μg/ml) |  |  |  |
| **HDK-20**  (0.4 μg/ml) |  |  |  |

**Figure S**2. TUNEL assay histogram plots of treated and untreated *T. brucei* s427 wt cells.

| **Mitochondrial membrane potential (%Ψm) of Untreated (control) *T.b.b* s427 WT cells** | | | |
| --- | --- | --- | --- |
| **Incubation time (h)** | **Experiment 1** | **Experiment 2** | |
| **1** |  |  | |
| **4** |  |  | |
| **8** |  |  | |
| **12** |  |  | |
| **Mitochondrial membrane potential (%Ψm) of treated *T.b.b* s427 WT cells with Troglitozone (10** **µM).** | | | |
| **1** |  |  | |
| **4** |  |  | |
| **8** |  |  | |
| **12** |  |  | |
| **Mitochondrial membrane potential (%Ψm) of treated *T.b.b* s427 WT cells with Valinomycin (100 nM).** | | | |
| **1** |  | |  |
| **4** |  | |  |
| **8** |  | |  |
| **12** |  | |  |
| **Mitochondrial membrane potential (%Ψm) of treated *T.b.b* s427 WT cells with HDK 20 (0.3 µg/ml)** | | | |
| **1** |  | |  |
| **4** |  | |  |
| **8** |  | |  |
| **12** |  | |  |

**Figure S3.** Histograms of mitochondrial membrane potential (%Ψm) of treated and untreated *T.b.b* s427 WT cells.
